# Reducing neonatal Fc receptor binding enhances clearance and brain-to-blood ratio of TfR-delivered bispecific amyloid-β antibody

**DOI:** 10.1080/19420862.2024.2339337

**Published:** 2024-04-18

**Authors:** Eva Schlein, Ken G. Andersson, Tiffany Dallas, Stina Syvänen, Dag Sehlin

**Affiliations:** a Department of Public Health and Caring Sciences, Uppsala University, Uppsala, Sweden; b Biotechnology, BioArctic AB, Stockholm, Sweden

**Keywords:** Alzheimer’s disease (AD), amyloid-β (aβ), bispecific antibody, blood-brain barrier (BBB), neonatal Fc receptor (FcRn), receptor mediated transcytosis (RMT)

## Abstract

Recent development of amyloid-β (Aβ)-targeted immunotherapies for Alzheimer’s disease (AD) have highlighted the need for accurate diagnostic methods. Antibody-based positron emission tomography (PET) ligands are well suited for this purpose as they can be directed toward the same target as the therapeutic antibody. Bispecific, brain-penetrating antibodies can achieve sufficient brain concentrations, but their slow blood clearance remains a challenge, since it prolongs the time required to achieve a target-specific PET signal. Here, two antibodies were designed based on the Aβ antibody bapineuzumab (Bapi) – one monospecific IgG (Bapi) and one bispecific antibody with an antigen binding fragment (Fab) of the transferrin receptor (TfR) antibody 8D3 fused to one of the heavy chains (Bapi-Fab8D3) for active, TfR-mediated transport into the brain. A variant of each antibody was designed to harbor a mutation to the neonatal Fc receptor (FcRn) binding domain, to increase clearance. Blood and brain pharmacokinetics of radiolabeled antibodies were studied in wildtype (WT) and AD mice (*App*^*NL-G-F*^). The FcRn mutation substantially reduced blood half-life of both Bapi and Bapi-Fab8D3. Bapi-Fab8D3 showed high brain uptake and the brain-to-blood ratio of its FcRn mutated form was significantly higher in *App*^*NL-G-F*^ mice than in WT mice 12 h after injection and increased further up to 168 h. *Ex vivo* autoradiography showed specific antibody retention in areas with abundant Aβ pathology. Taken together, these results suggest that reducing FcRn binding of a full-sized bispecific antibody increases the systemic elimination and could thereby drastically reduce the time from injection to *in vivo* imaging.

## Introduction

Recent developments in protein engineering, combined with the high affinity and specificity of antibodies has promoted the growth of biological molecules in pharmaceutical applications. Previously, antibody drugs have mainly been directed to peripheral targets, such as various forms of malignancies. Only recently have the first antibodies for neurodegenerative diseases reached the market after the US Food and Drug Administration approval of lecanemab,^1–3^ and aducanumab,^4,5^ which both target amyloid-β (Aβ) in Alzheimer’s disease (AD). Therapies directed toward specific pathologies require reliable diagnostic methods to select patients that express the intended target. The most advanced diagnostic method now used for inclusion of patients for Aβ-directed therapies is positron emission tomography (PET) using amyloid ligands, such as [^11^C]PiB. ^6–8^ However, the currently available amyloid ligands detect only the dense core of amyloid plaques and not the soluble or diffuse Aβ aggregates that are targeted by therapeutic antibodies, such as lecanemab and aducanumab.^8–10^ Thus, a more exact identification of patients to include for therapy could be achieved if these therapeutic antibodies could also be used as PET imaging ligands.^10,11^

With antibody-based PET radioligands, the design of a diagnostic and therapeutic antibody pair could be an attractive strategy, as the diagnostic version of the antibody, i.e., the antibody-based radioligand, could be used for patient inclusion, to assess target engagement and to evaluate therapeutic effects. However, some challenges must be addressed to reach this goal. First, antibody transport into the brain is slow and inefficient. For chronic applications, the steady albeit slow influx of antibody into the brain has proven enough for the therapeutic antibody to have an Aβ-reducing effect,^3,11,12^ but imaging is based on a single injection of the radioligand that must reach high brain concentrations rapidly. For antibody-based PET radioligands, this can be achieved with a molecular Trojan horse strategy that takes advantage of transferrin receptor (TfR)-mediated transcytosis to ferry proteins across the blood–brain barrier (BBB) and into the brain.^13,14^ Antibodies engineered into a bispecific format to enable binding to TfR display an increased brain uptake and have been used for both therapeutic^15–18^ and imaging purposes.^19,20^ The mode of TfR binding appears to be important for the efficiency of such bispecific antibodies to enter the brain, favoring mono- over bivalent TfR interaction, to avoid cell surface receptor clustering and sorting for lysosomal degradation.^21,22^ For an asymmetrical IgG antibody design, a monovalent TfR interaction is generally achieved by using a knobs-into-holes design, where the TfR binding moiety can be incorporated either as one of the antibody halves,^23^ as a moiety fused to one of the antibody’s heavy or light chains,^21^ or as a specific TfR-binding amino acid sequence within one of the heavy chain constant regions.^18^

A second challenge is the long biological half-life of antibodies, which is an advantage in the therapeutic setting, but a challenge for *in vivo* imaging applications, where rapid clearance of unbound antibody from both blood and brain is required to achieve high imaging contrast. The circulation of IgG antibodies in blood is regulated by the neonatal Fc receptor (FcRn), which rescues the antibodies from lysosomal degradation.^24^ If this maintenance mechanism is inhibited, for example by a genetic loss of the FcRn function, the IgG levels in blood can be markedly reduced through increased catabolism by macrophages.^25^ FcRn has also been reported to be involved in reverse gatekeeping, mediating active efflux of IgG over the BBB and out of the brain.^26–30^ Modulation of antibody interactions with FcRn has been explored for tumor-imaging^31,32^ and therapeutic applications,^33–35^ but its relevance for brain PET imaging has not been explored yet. Thus, reduced binding to the FcRn could be beneficial for a full IgG immunoPET ligand, as a shorter biological half-life could reduce unspecific signal derived from the blood volume of the brain. Another aspect in which therapeutic and diagnostic antibody applications differ is the antibody’s effector functions, which, to a large degree, is mediated by interactions of the antibody Fc domain with Fc receptors on immune cells. These interactions can be crucial for therapy as part of the mechanism of action, but for diagnostic applications such as PET imaging, minimal interactions with the immune system is desired. This can be accomplished by introduction of mutations to silence an antibody’s interactions with Fc receptors.^36^

Here, we describe the design and *in vivo* evaluation of a brain-penetrating bispecific Aβ antibody with impaired FcRn binding aimed to reduce the biological circulation time of the antibody, with the long-term goal to develop a selective ligand for Aβ immunoPET imaging.

**Table 1.** Number of animals.

Antibody	Number of injected animals (WT/*App*^*NL-G-F*^) per antibody and time point				
	*3 h*	*12 h*	*24 h*	*72 h*	*168 h*
Bapi-Fab8D3	4/-	4/3	4/4	3/3	7/3
Bapi-Fab8D3^FcRn-^	4/-	4/3	3/4	3/3	7/3
Bapi	4/-	-/-	-/-	3/3	4/-
Bapi^FcRn-^	4/-	-/-	-/-	3/3	4/-

## Results

**Figure 1.**
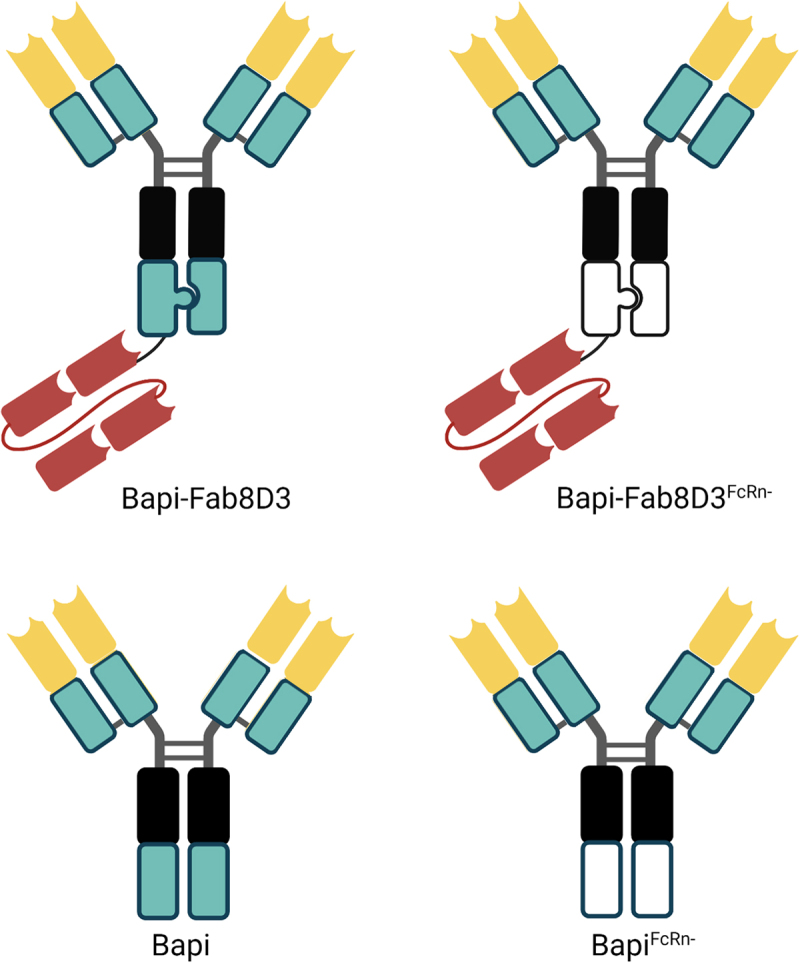
Schematic visualization of the antibody design. All antibodies were based on the variable domains of Bapi in yellow, with the addition of a Fab8D3 fragment in red for bispecific variants. A black box represents mutations in the CH2, reducing the effector functions; knobs-into-holes design is visually represented by a knob and a corresponding hole; a green box represents non-mutated constant region whereas a white box indicates FcRn mutation in CH3.

The antibodies used in this study were based on the humanized monoclonal antibody bapineuzumab (Bapi)^38^ that binds specifically to the N-terminus of Aβ (amino acid 1–5). To generate bispecific antibodies with monovalent binding to the mouse TfR (mTfR), constructs were designed based on the initial design of the Roche brain shuttle format,^21^ with a single-chain antigen binding fragment (scFab) of the TfR antibody 8D3^39^ attached via a linker to the C-terminus of one of the Bapi heavy chains. The design of this scFab variant was based on the knobs-into-holes technique reported in patent WO2014033074A1 (SEQ_ID 1; IgG1),^40^ with Bapi heavy and light variable region sequences replacing the original variable sequences. Monospecific Bapi IgG was produced for comparison. Both antibody formats were designed with and without mutations that reduce binding to the FcRn (H310A, H435Q),^31^ referred to as *FcRn-*. In addition, effector function reducing mutations (L234A, L235A, P329G)^41^ were introduced at the Fc domain of all antibodies. A monovalent Bapi, without mutations, was produced as a control. The antibody design is summarized in Figure 1.

**Figure 2.**
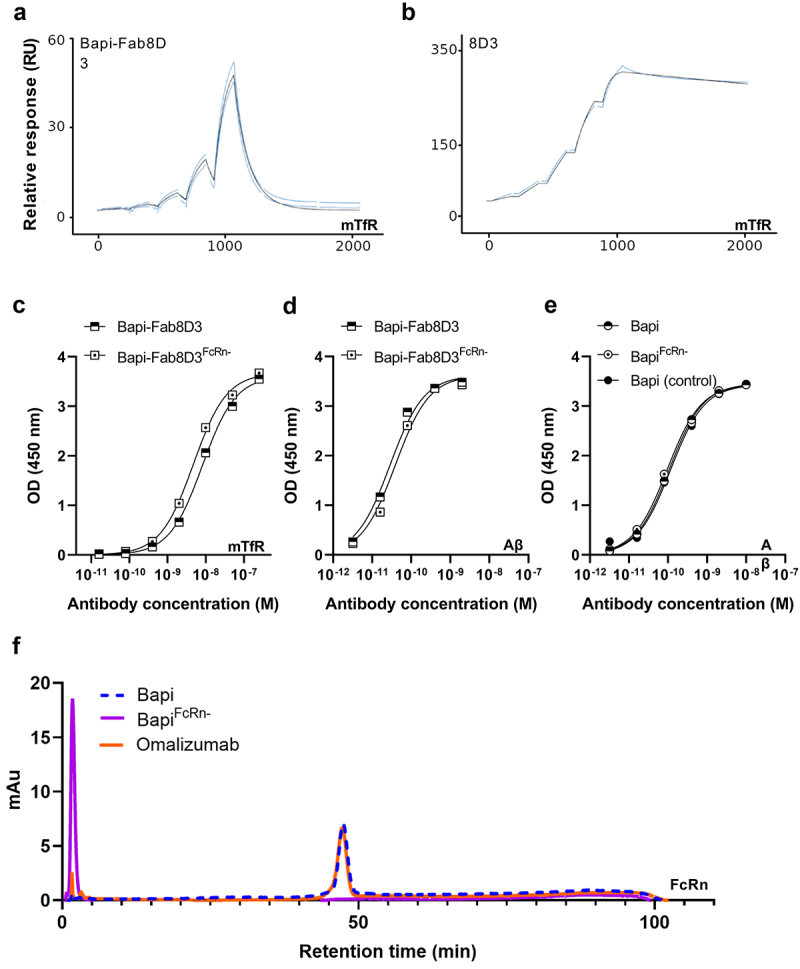
Quality control of the antibody constructs. A. Representative Biacore sensograms displaying monovalent TfR interaction of Bapi-Fab8D3 constructs. B. Representative Biacore sensograms displaying bivalent TfR interaction of 8D3 IgG. C. TfR ELISA analysis of bispecific antibody constructs D. Aβ ELISA analysis of bispecific antibody contructs E. Aβ ELISA analysis of monospecific antibody contructs F. Representative chromatogram of FcRn-column analysis of Bapi^FcRn-^ (purple), Bapi (blue) and positive control Omalizumab (orange).

All antibodies were produced with a good yield, although the yield was somewhat lower for bispecific variants. Furthermore, all antibodies were at least 97% monomeric (Table 2). To confirm the monovalent TfR binding of the bispecific antibody constructs (Bapi-Fab8D3 and Bapi-Fab8D3^FcR-^), Biacore surface plasmon resonance (SPR) analysis was conducted. Sensorgram analysis revealed a strict monovalent binding interaction to mTfR of both bispecific antibodies (Figure 2a). The 8D3 IgG control showed a typical bivalent interaction pattern (Figure 2b), whereas the Bapi variants did not display any binding to TfR. While SPR analysis showed a 3–4 fold higher K_D_ value than previously reported for Fab8D3 alone,^42^ the TfR ELISA analysis was in line with previous studies on this bispecific antibody format (Figure 2c).^21^ Further, ELISA was also used to assess the antibodies’ binding affinity to Aβ (Figure 2d–e and Table 2). To investigate the impact of the FcRn-attenuating mutation introduced to a subset of the antibodies, their interaction with FcRn protein, immobilized on a column, was analyzed in comparison with the humanized IgG1 antibody omalizumab. A retention time of 1.7 minutes was recorded for FcRn mutated antibodies, whereas non-mutated antibodies and omalizumab displayed a retention time of 46.4 minutes, indicating that non-mutated antibodies bound to FcRn with equal affinity as the control, whereas mutants had markedly lowered affinity to FcRn (Figure 2f and Table 2).

**Table 2.** Detailed description of antibody design and properties.

Antibody	Yield (mg/L culture)	Purity (% monomer)	mTfR affinity (KD; M)	Aβ affinity (EC50; M)	FcRn ret. time (min)
Bapi-Fab8D3	18.3*	97.9	1.09e-07	1.3e-10	46.4
Bapi-Fab8D3^FcRn-^	19.4*	99.7	1.18e-07	6.6e-11	n.d.
Bapi	29.2	96.8	n.a.	1.4e-10	46.4
Bapi^FcRn-^	35.7	99.3	n.a.	1.4–10	1.7
Bapi (control)**	36.8	99.9	n.a.	1.2e-10	46.4

*purified with a two-step method. **control variant without mutations.

**Figure 3.**
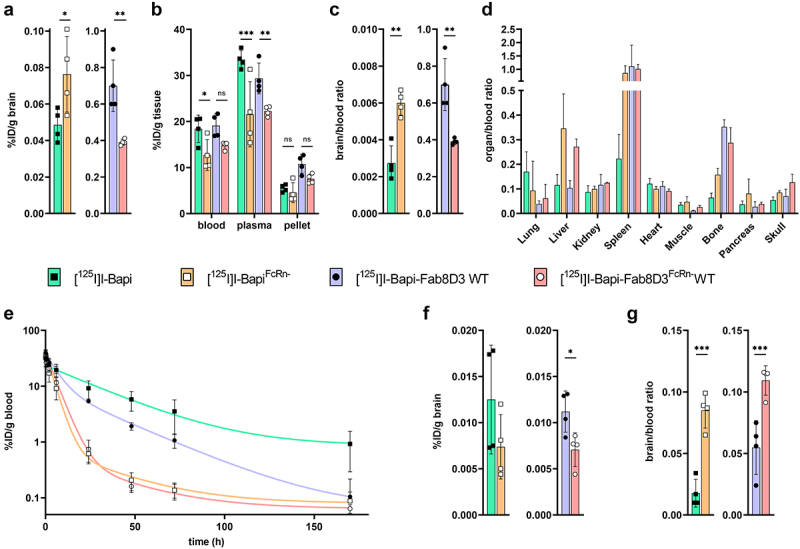
*Ex vivo* analysis of antibody distribution in WT mice. A. Antibody retention in the brain, expressed as percent of injected dose per gram (%ID/g) tissue, 3 h after injection of [^125^I]I-Bapi, [^125^I]I-Bapi^FcRn-^, [^125^I]I-Bapi-Fab8D3 and [^125^I]I-Bapi-Fab8D3^FcRn-^ (*n* = 4 per antibody). B. Blood, plasma and blood cell pellet concentration (%ID/g) 3 h after injection. C. Brain-to blood ratio 3 h post injection. D. Biodistribution, expressed as organ-to-blood concentration, 3 h after injection. E. Total blood concentration (%ID/g) of the four antibodies over a time course of seven days (*n* = 3–4 per antibody) with two-phase decay curve fit. F. Brain concentration (%ID/g) 7 days after injection. G. Brain-to-blood ratio seven days after injection.

To study the effect of the FcRn mutation *in vivo*, Bapi, Bapi^FcRn-^, Bapi-Fab8D3, and Bapi-Fab8D3^FcRn-^ were radiolabeled with iodine-125 (^125^I) and injected into wild-type (WT) mice. First, brain uptake and biodistribution were evaluated at 3 h after antibody injection. As expected, brain retention of the bispecific variants was higher than for the Bapi variants. Interestingly, while [^125^I]I-Bapi^FcRn-^ showed higher brain uptake than its non-mutated variant, the brain concentration of [^125^I]I-Bapi-Fab8D3^FcRn-^ was significantly lower than [^125^I]I-Bapi-Fab8D3 (Figure 3a). FcRn mutated antibodies also displayed lower blood and plasma concentrations compared with their non-mutated variants (Figure 3b). Still, the relative brain uptake, expressed as the brain-to-blood ratio, was lower for [^125^I]I-Bapi-Fab8D3^FcRn-^ compared with [^125^I]I-Bapi-Fab8D3 (Figure 3c). The bispecific antibodies showed a high distribution to the spleen and bone, as a result of TfR binding in these tissues. Moreover, [^125^I]I-Bapi^FcRn-^ also displayed high spleen accumulation (Figure 3d). In addition, both mutated antibody variants had increased accumulation in the liver, indicative of degradation (Figure 3d).

To investigate the pharmacokinetic properties of the antibodies, their blood concentrations were measured in WT mice over a period of seven days after injection (Figure 3e). Antibodies with the FcRn mutation displayed a markedly increased clearance, compared with their non-mutated variants, visible already 6 h post injection and with a clear separation after 24 h. Interestingly, although the non-mutated [^125^I]I-Bapi-Fab8D3 displayed faster clearance than [^125^I]I-Bapi, due to its interaction with TfR,^43,44^ the two mutated variants had very similar pharmacokinetics over seven days, with a half-life of 4.5 h (Figure 3e). Seven days after injection, the brain retention of all antibodies was low, without significant differences between the [^125^I]I-Bapi variants but with slightly higher brain concentration of [^125^I]I-Bapi-Fab8D3 compared with [^125^I]I-Bapi-Fab8D3^FcRn-^ (Figure 3f). However, the relative brain retention, expressed as the brain-to-blood concentration ratio, was higher for both antibodies with the FcRn mutation, compared to the antibodies without the mutation (Figure 3f).

**Figure 4.**
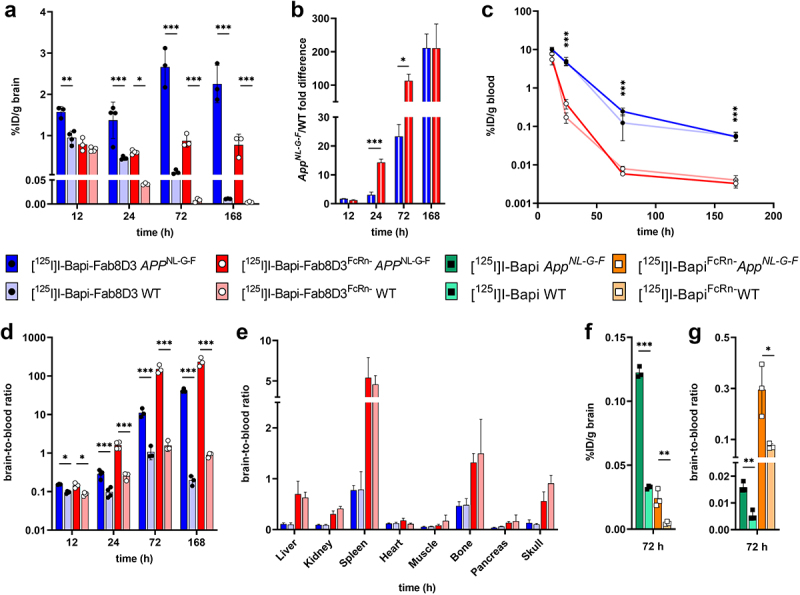
*Ex vivo* analysis of antibody distribution in *App*^*NL-G-F*^ compared to WT mice. A. Brain retention (%ID/g) of [^125^I]I-Bapi-Fab8D3 and [^125^I]I-Bapi-Fab8D3^FcRn-^ at 12 h−168 h after injection. B. Fold difference in brain retention (*App*^*NL-G-F*^/WT) at 12 h−168 h after injection of [^125^I]I-Bapi-Fab8D3 and [^125^I]I-Bapi-Fab8D3^FcRn-^. C. Terminal blood concentration of [^125^I]I-Bapi-Fab8D3 and [^125^I]I-Bapi-Fab8D3^FcRn-^ in WT and *App*^*NL-G-F*^ mice at 12 h−168 h after injection. D. Brain-to-blood ratio of [^125^I]I-Bapi-Fab8D3 and [^125^I]I-Bapi-Fab8D3^FcRn-^ at 12 h−168 h after injection. E. Biodistribution to peripheral organs (organ-to-blood ratio) at 24 h after injection. F. Brain retention (%ID/g) of [^125^I]I-Bapi and [^125^I]I-Bapi^FcRn-^ at 72 h after injection. G. Brain-to-blood ratio of [^125^I]I-Bapi and [^125^I]I-Bapi^FcRn-^ at 72 h after injection.

Further studies focused on antibody brain uptake in the AD mouse model *App*^*NL-G-F*^ in comparison with WT mice. First, the bispecific Bapi-Fab8D3 and Bapi-Fab8D3^FcRn-^ were administered to *App*^*NL-G-F*^ and WT mice that were sacrificed at 12 h, 24 h, 72 h, and 168 h after injection. At 12 h after injection, brain retention of [^125^I]I-Bapi-Fab8D3 was about twice as high as that of [^125^I]I-Bapi-Fab8D3^FcRn-^ in *App*^*NL-G-F*^ mice. Further, while [^125^I]I-Bapi-Fab8D3 increased in the brain over time, the mutated [^125^I]I-Bapi-Fab8D3^FcRn-^ remained stable (Figure 4a). In WT mice that lack brain Aβ pathology, i.e., the antibodies' primary target, brain retention of both antibodies decreased over time, with a faster decline for [^125^I]I-Bapi-Fab8D3^FcRn-^. Thus, the difference between *App*^*NL-G-F*^ and WT mice increased at a faster rate for the mutated [^125^I]I-Bapi-Fab8D3^FcRn-^ compared to [^125^I]I-Bapi-Fab8D3 over the three first days after injection (Figure 4). Blood concentration of the antibodies (Figure 4c) largely followed the same pattern as previously seen in WT mice (Figure 3e), with significantly lower concentration of the mutated antibody at all time points. At 12 h after injection, the brain-to-blood ratio was similar for [^125^I]I-Bapi-Fab8D3 and [^125^I]I-Bapi-Fab8D3^FcRn-^, both displaying a 1.7-fold difference between *App*^*NL-G-F*^ and WT mice. Over the following three days, this ratio increased substantially more for the mutated antibody (Figure 4d). The antibodies’ distribution to peripheral organs was dominated by TfR-mediated retention in spleen, bone, and skull. In addition, [^125^I]I-Bapi-Fab8D3^FcRn-^ showed high concentration in liver and kidney, indicating degradation and secretion of free iodine (Figure 4e). For comparison, brain uptake of [^125^I]I-Bapi and [^125^I]I-Bapi^FcRn-^ was studied in *App*^*NL-G-F*^ and WT mice at 72 h after administration, i.e., the time point where the highest brain retention was seen for the bispecific antibody variants. Both antibodies showed a significantly higher brain retention in *App*^*NL-G-F*^ compared to WT mice and while [^125^I]I-Bapi displayed substantially higher absolute brain concentrations (Figure 4f), [^125^I]I-Bapi^FcRn-^ showed a higher brain-to blood ratio in both *App*^*NL-G-F*^ and WT mice (Figure 4g).

**Figure 5.**
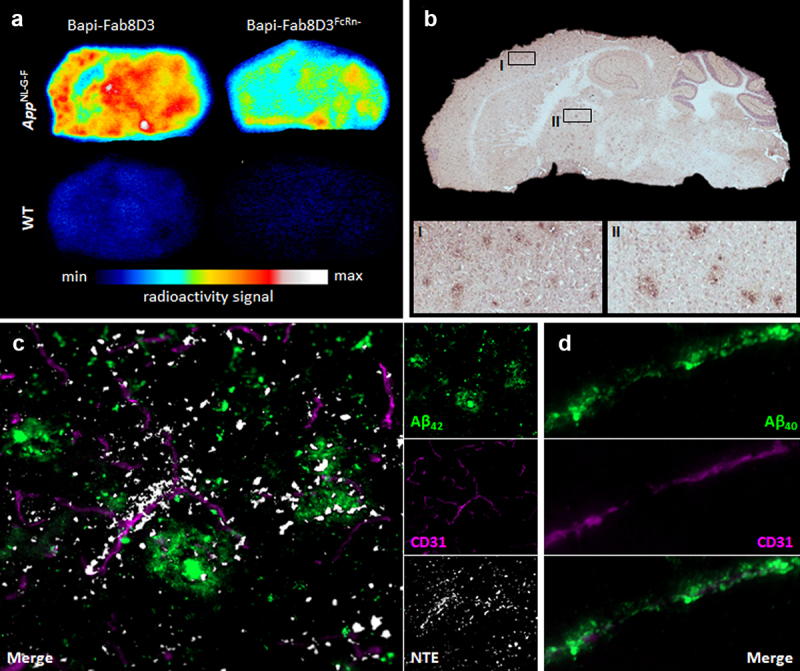
*Post mortem* analyses of antibody brain retention 24 h after injection. A. Representative images of *ex vivo* autoradiography illustrating the distribution of [^125^I]I-Bapi-Fab8D3 and [^125^I]I-Bapi-Fab8D3^FcRn-^ in sagittal brain sections from *App^NL-G-F^* and WT mice at 24 h after antibody injection. B. Immunostaining of total Aβ (3D6) in *App^NL-G-F^* brain with squares indicating magnified areas of abundant Aβ pathology in cortex (I) and thalamus (II). C. Nuclear track emulsion autoradiography (NTE; white puncta) in combination with immunofluorescent staining of Aβ_42_ (green) and endothelial cell marker CD31 (pink), 24 h after injection of [^125^I]I-Bapi-Fab8D3^FcRn-^ in *App^NL-G-F^* mice, showing antibody retention along vessels and around Aβ deposits. D. Immunofluorescent staining of *App^NL-G-F^* mouse brain with CD31 (pink) and Aβ_40_ (green) demonstrating abundant Aβ_40_ deposition along a brain vessel.

To visualize antibody distribution in the brain, *ex vivo* autoradiography was carried out 24 h after injection of [^125^I]I-Bapi-Fab8D3 and [^125^I]I-Bapi-Fab8D3^FcRn-^. This analysis confirmed results from the *ex vivo* study, with a higher overall signal from [^125^I]I-Bapi-Fab8D3 compared to [^125^I]I-Bapi-Fab8D3^FcRn-^ in both *App*^*NL-G-F*^ and WT brain (Figure 5a). Both antibodies were distributed primarily to the cortex and thalamus, i.e., areas of abundant Aβ pathology in the *App*^*NL-G-F*^ model (Figure 5b). [^125^I]I-Bapi-Fab8D3^FcRn-^ distribution was studied at high resolution in the *App*^*NL-G-F*^ brain at 24 h after injection with NTE autoradiography combined with immunostaining of Aβ and the vasculature (Figure 5c). The antibody was found in close proximity to both Aβ deposits and brain vessels. Although the perivascular retention of [^125^I]I-Bapi-Fab8D3^FcRn-^ could not be attributed to specific interaction with Aβ42, separate double staining of the brain tissue suggested accumulation Aβ40 along brain vessels in the *App*^*NL-G-F*^ mouse (Figure 5d).

## Discussion

The recent progress in AD immunotherapy has been an important milestone in the development of antibody drugs for brain applications. While the poor brain penetration of antibodies remains a challenge, clinical use of brain shuttles to ferry protein drugs into the brain is already emerging.^45^ This development will likely improve the effectiveness of biologic drugs designed to be active in the brain. Additionally, it introduces the prospect of using antibodies as *in* *vivo* imaging ligands for brain targets, potentially via therapeutic antibodies that are modified to function as diagnostic companions, thereby augmenting the precision of therapy. Here, we explored a mutation that reduces antibody interaction with the FcRn to increase blood clearance of the bispecific antibody Bapi-Fab8D3, aiming to increase the brain-to-blood contrast and enable immunoPET imaging on the same day as antibody injection. Indeed, the FcRn mutation markedly increased clearance of both the monospecific antibody Bapi^FcRn-^ and its bispecific variant Bapi-Fab8D3^FcRn-^, which showed nearly identical blood pharmacokinetic profiles over seven days after injection in WT mice.

Bapi-Fab8D3, designed for monovalent TfR binding using the knobs-into-holes technique, displayed the expected binding to Aβ and TfR, resulting in good brain penetration compared with the regular Bapi. Interestingly, Bapi-Fab8D3 displayed significantly higher brain uptake compared with Bapi-Fab8D3^FcRn-^ at 3 h after injection, both in absolute concentration and when expressed as a brain-to-blood concentration ratio (Figure 3a & 3c). However, at later time points, Bapi-Fab8D3^FcRn-^ displayed a higher brain-to-blood ratio in WT mice, which lack the antibodies’ target in the brain (Figure 4d). This deviation is probably mainly caused by the rapid clearance of Bapi-Fab8D3^FcRn-^ from blood. It has been suggested that antibody efflux from the brain is mediated by FcRn. While the faster increase in brain-to-blood ratio of Bapi-Fab8D3^FcRn-^ could indicate a slower clearance from the brain, this effect is probably quite marginal and is not reflected by the absolute brain concentrations over time (Figure 4a). The lower brain uptake of Bapi-Fab8D3^FcRn-^ is likely due to its rapid blood clearance that leads to decreased exposure, which to some degree prevents interaction with TfR at the BBB. It could also indicate that TfR-mediated transcytosis is boosted by a synergistic effect if the antibody also interacts with FcRn, which has been suggested to promote transcytosis on its own. A recent study proposed a bidirectional nature of the FcRn expressed at the BBB. By increasing the binding to the FcRn at pH 6 and 7.4 using the YTE substitution at the Fc domain, antibodies were transported into the brain.^46^ When introducing the FcRn-ablating mutation to the monospecific antibody Bapi, a significant increase in brain uptake was observed at 3 h after injection, both in absolute numbers and even more pronounced in relation to blood concentration. Although brain concentrations were generally very low, the higher relative brain concentration of Bapi^FcRn-^ compared to Bapi in WT mice increased further at three and seven days after antibody administration (Figure 3g & 4g). Again, absolute brain concentrations were substantially lower for Bapi^FcRn-^ (Figure 4f), and these observations do not fully support a reduced FcRn-mediated efflux from the brain. Any potential discrepancies in how the mono- and bispecific antibody variants react to FcRn mutations could be due to their different brain entry routes – while bispecific antibodies are distributed to the brain parenchyma by TfR-mediated transcytosis through the endothelium of the widespread net of brain capillaries, standard monospecific antibodies have been suggested to mainly reach the brain through perivascular transport along larger brain penetrating vessels.^47^ It should also be noted that the binding of human Fc to mouse FcRn is stronger than to human FcRn.^48^ Results obtained in mice should therefore be interpreted with some caution regarding translation to humans.

All antibodies displayed specific retention in the brain of *App*^*NL-G-F*^ mice that express Aβ (Figure 4). This was demonstrated both as a higher total antibody concentration associated with the brain in *App*^*NL-G-F*^ mice compared with WT mice, but also with *ex vivo* autoradiography that visualized the regional brain distribution of the bispecific antibodies. Interestingly, [^125^I]I-Bapi-Fab8D3^FcRn-^ distribution in *App*^*NL-G-F*^ brain studied with NTE at high magnification revealed that the antibody was found around parenchymal Aβ deposits and also in close proximity to the vasculature. Although Aβ42, the predominant Aβ isoform in *App*^*NL-G-F*^ mice,^49^ could not be found in the vasculature, a clear Aβ40 staining was seen along brain vessels, suggesting that Bapi-Fab8D3, which does not discriminate between Aβ40 and Aβ42, can detect Aβ deposits of different structure (Figure 5). In addition, no specific antibody retention was seen in WT mice, suggesting that the brain retention observed 24 h after injection of [^125^I]I-Bapi-Fab8D3^FcRn-^ is indeed specific to Aβ, which is only expressed in the *App*^*NL-G-F*^ mice.

Previous studies have shown that bispecific full-length IgG antibodies targeting Aβ can discriminate between AD and WT mice with PET imaging several days after injection.^13,50,51^ We show here that both Bapi-Fab8D3 and Bapi-Fab8D3^FcRn-^ display higher total brain concentrations than our previously studied bispecific antibodies. In addition, distinctions between AD and WT mice were clear at 24 h after injection. These improvements could be due to the monovalent TfR interaction that increases brain uptake and reduces interaction with vascular and parenchymal TfR, which could promote faster clearance from the brain.^21,52^ The total brain concentration was higher for Bapi-Fab8D3, which suggests that this antibody could be a good candidate for pretargeted PET imaging, using biorthogonal click chemistry,^53^ where the most important antibody feature is a high brain concentration. However, the difference between *App*^*NL-G-F*^ and WT mice was substantially larger for Bapi-Fab8D3^FcRn-^, which in addition displayed a greater brain-to-blood ratio than Bapi-Fab8D3 (Figure 4b & 4d). Higher brain-to-blood ratio, in combination with low absolute levels of antibodies in the blood, are key factors for successful high-contrast immunoPET imaging of a brain target.^11,14,54^ When comparing the blood pharmacokinetics of Bapi-Fab8D3 and Bapi-Fab8D3^FcRn-^ over time, it was evident that the blood concentrations of Bapi-Fab8D3^FcRn-^ were low already at 12 h after injection. Bapi-Fab8D3^FcRn-^ also displayed a significantly higher brain-to-blood ratio in *App*^*NL-G-F*^ compared to WT mice at 12 h. Although this is not an ideal time point for PET imaging, it is substantially closer to same-day imaging compared with the 3- to 4-day period that has previously been the standard for immunoPET imaging of Aβ.^13,14,50,51,55^ Mutations to the FcRn binding site of a full-length antibody thus provides a significant improvement and a potential tool to enable immunoPET imaging with therapeutic antibodies, without major changes to their structure.

## Materials and methods

### Antibody design and production

All antibody constructs were synthesized using GeneArt (Thermo Fisher Scientific, Regensburg, Germany) and cloned into pcDNA3.4 vector, then expressed recombinantly in ExpiCHOTM cells (Gibco^TM^, Cat. A29127) using the ExpiCHO Expression System Kit (Thermo Fisher, Cat. A29133). The pcDNA3.4 vectors encoding the heavy and light chains were added to the cells and transfection was performed according to the manufacturer’s instructions. Cells were cultured in ExpiCHOTM Expression medium in a humidified incubator with 8% CO2 at 37 °C, 120 rpm for 7–9 days, and then centrifuged at 18 000 rpm for 40 minutes. The antibody-containing cell supernatant was sterile filtered using a 0.22 µM filter (Corning) and the antibodies were subsequently purified from the cell supernatant with a HiTrap MabSelect SuRe (Cytiva, Cat. 11-0034-93) column using an ÄKTA system. The supernatant was loaded on to the MabSelect SuRe column, followed by washing with 5 column volumes of phosphate-buffered saline (PBS; Gibco) and elution with a linear gradient of 0.7% acetic acid. For antibodies without an mTfR binding moiety, the buffer was changed to PBS using a HiPrep 26/10 desalting column (Cytiva, Uppsala, Sweden), subsequent to purification. Bispecific antibodies designed with knobs-into-holes mutations were further purified with size exclusion chromatography (SEC) using a HiLoadⓇ 16/600, SuperdexⓇ 200 pg column. The peak containing monomeric antibody was collected.

### Quality control

For quality control of antibody size and purity, analytical SEC was carried out using a TSKgelⓇ3000GSWxl size column (7.8 × 300 mm, 5 μm particle size; Tosoh Bioscience) and an Agilent HPLC 1100 system (Agilent Technologies, California, U.S.) with a flow rate of 0.5 mL/min. A total of 10 µg of antibody was injected onto the column and the residence time as well as the monomeric content was analyzed using data analysis software (Agilent Technologies). The antibodies containing an mTfR binding module were analyzed for the presence of endotoxin by using EndosafeⓇ-PTS cartridge in the EndosafeⓇ nexgen-PTS ^TM^ system according to manufacturers’ instruction (Charles River, Massachusetts, U.S.).

### Biacore analysis

The binding kinetics of the antibodies to mTfR was determined by surface plasmon resonance (SPR) using Biacore 8K (Cytiva, Uppsala, Sweden). The TfR antibody 8D3 was used as positive control for bivalent TfR binding. To prepare the analysis, 1.5 µg/mL of mTfR (in-house produced) in NH_4_Ac pH 5.5 (Cytiva) was immobilized on eight surfaces of a CM5 chip (Cytiva) using amine-coupling chemistry NHS/EDC according to manufacturer’s instruction. A single cycle kinetic analysis was performed by injecting duplicates of five 1:2 dilutions of antibody construct with a starting concentration of 50 nM. The surface was regenerated using 3 M NaCl between injections. Biacore Insight evaluation software (Cytiva, Uppsala, Sweden) was used to fit data to the sensorgram using a 1:1 kinetic fit model.

### ELISA

TfR and Aβ ELISA binding assays, as well as an anti-human IgG sandwich-ELISA for antibody quantification after radiolabeling, were performed as previously described.^14^ (Jackson ImmunoResearch Laboratories, West Grove, PA, U.S.), and signals were developed with K Blue Aqueous TMB substrate (Neogen Corp., Lexington, KY, U.S.) and read with a spectrophotometer at 450 nm. All dilutions were made in ELISA incubation buffer (PBS, 0.1% BSA, 0.05% Tween-20).

### FcRn affinity chromatography

Analytical FcRn affinity chromatography was carried out using an FcRn affinity column (Roche Diagnostics GmbH, Mannheim, Germany) on an Agilent HPLC 1100 system (Agilent Technologies). Two buffers were prepared, *buffer A*: 20 mM MES, 140 mM NaCl pH 5.5 and *buffer B*: 20 mM Tris, 140 mM NaCl pH 8.8. A 30 µg sample of each antibody construct, including the humanized IgG antibody omalizumab as positive control for FcRn binding, underwent buffer exchange into buffer A. Each antibody was then loaded onto the column analyzed with a flow rate of 0.5 mL/min. An initial 10-minute isocratic flow of 20% *buffer B* was followed by a 20–100% *buffer B* gradient over 80 minutes. After elution of the sample, the column was re-equilibrated for 13 minutes with 20% buffer B.

### Radiolabeling

For ex vivo studies, the antibodies were radiolabeled with iodine-125 (125I) using the chloramine T method, as previously described.^37^I (PerkinElmer Inc., Waltham, MA, U.S.). Chloramine-T (Sigma Aldrich) was added (5 µg, 200 µM in PBS) and incubated for 90 seconds at room temperature. To quench the reaction, sodium-metabisulfite (10 µg, 440 µM in PBS, Sigma Aldrich) was added. To purify the radiolabeled antibody from remaining excess of I, a disposable Zeba spin desalting column was used (7K MWCO, 0.5 mL, ThermoFisher). The final activity of the purified product was measured in an ion chamber.

### Animal studies

Animal studies were conducted in 14–16 month old female and male wild-type (WT) or App^*NL-G-F*^ mice maintained on a C57BL/6 genetic background. App*^NL-G-F^* mice express human APP with three mutations – KM670/671NL (Swedish), E693G (Arctic), and I716F (Iberian), resulting in accumulation of Aβ from the age of two months.^49^ The animals were housed with unlimited access to food and water in rooms with controlled temperature and humidity in an animal facility at Uppsala University. All procedures described were approved by the Uppsala County Animal Ethics Board (5.8.18–20401/2020) and were in accord with the rules and regulations of the Swedish Animal Welfare Agency and complied with the European Communities Council Directive of 22 September 2010 (2010/63/EU).

For *ex vivo* studies, the radiolabeled antibodies were injected into the tail vein of mice under mild isoflurane anesthesia (Baxter Medical AB, Kista, Sweden) at a dose of 1.6 nmol/kg body weight. For blood kinetic experiments, blood samples were obtained with 8 µL capillaries at 5 min, 30 min, 2 h, 6 h, 24 h, 48 h, and 72 h post-injection and a terminal blood sample was taken from the heart before animals were euthanized by intracardiac perfusion with 0.9% saline over 3 min. Brain and peripheral organs (lung, liver, kidney, spleen, heart, muscle, femoral bone, pancreas, skull, thyroid, plasma, and blood pellet) were isolated from WT and App*^NL-G-F^* mice at 3 h, 12 h, 24 h, 72 h, or 168 h after injection to assess the tissue concentrations of the radiolabeled proteins over time. The brain was divided into the right and left hemispheres, with the left hemisphere further divided into brain and cerebellum. Radioactivity was measured in blood and organs using a γ-counter (2480 Wizard, PerkinElmer, Waltham, U.S.) and concentrations were expressed as % of injected dose per gram tissue (% ID/g). Number of injected mice is given in Table 1.

### Immunostainings and nuclear track emulsion

Immunohistochemistry was used to visualize both the Aβ pathology and the anatomical structure of the mouse brain tissue. Sagittal cryosections of 20 µm were fixed for 20 min in 4% formaldehyde and washed in PBS. Antigen retrieval was then performed by incubating the slides in a pre-heated citrate buffer (25 mM, pH 7.3), and then in 70% formic acid. Endogenous peroxidases were blocked with 3% hydrogen peroxide in PBS for 20 min. Unspecific binding was blocked with mouse on mouse (MOM) IgG blocking reagent (Vector, Catalog no. BMK 2202) for 1 h at room temperature (RT) with shake (100–200 rpm). Tissue sections were then permeabilized with 0.4% triton X-100 in PBS for 5 min with shake at 100–200 rpm. Following permeabilization, slides were incubated with MOM mouse diluent for 5 min at RT with shake (100–200 rpm), and then with Aβ antibody 3D6 (0.01 mg/mL in MOM diluent with 1×PBS +0.1% Tween20) overnight at +4°C with shake (100–200 rpm). Subsequently, slides were incubated with secondary antibody goat-anti-mouse-biotin (Vector Laboratories Inc., Burlingame, CA) diluted 1:250 in PBS, followed by incubation with streptavidin-HRP (Mabtech AB, Nacka Strand, Sweden) diluted 1:500 in PBS; both incubations occurring for 45 min at RT and shake 100–200 rpm. Color development was processed with Nova Red chromogen (Vector Laboratories Inc.). Slides were then counterstained with hematoxylin for 3 seconds and dehydrated in ascending concentrations of ethanol (70% to 96% to 2 × 99.9%) before being dipped in xylene. Slides were then mounted in mounting media Pertex overnight at RT and imaged with a Zeiss Observer Z.1 microscope using ZEN 3.7 software (Carl Zeiss Microimaging GmbH, Jena, Germany).

Nuclear track emulsion (NTE) was performed to analyze the retention of the 125I radiolabeled bispecific antibodies in brain tissue in relation to vessels and Aβ pathology. In short, sagittal cryosections of 20 µm were fixed for 10 min in ice-cold methanol, then permeabilized with 0.4% Triton-X in PBS for 10 min. Unspecific binding was blocked with 5% normal goat serum for 1 h at RT. Tissue sections were then permeabilized in 0.1% Tween-20 in PBS for 5 min and incubated with primary antibodies rat-anti-mouse CD31 (1.25 μg/mL; BD Biosciences), rabbit anti-Aβ40 (1 µg/ml; Agrisera, Umeå, Sweden, custom production) or rabbit anti-Aβ42 (1 µg/ml; Invitrogen, Waltham, MA), in 0.1% Tween-20 in PBS overnight at +4°C. Tissue sections were then incubated for 1 h at RT with secondary antibodies, Alexa-647-conjugated goat anti-rat IgG (1:200, Invitrogen) and Alexa-488-conjugated goat anti-rabbit IgG (1:200 of a 2 mg/mL stock, Invitrogen) in PBS. Following secondary antibody incubation, a subset of slides underwent NTE autoradiography. Sections were then dipped into melted Ilford K5 emulsion for 5 s and left to air-dry for 2 h at RT in the dark, then stored at +4°C for 4 weeks to expose the emulsion to the radioactive tissue. Tissue sections were developed according to manufacturers’ instructions. After development, the slides were air-dried and then mounted with Pertex overnight. Immunofluorescence and NTE were imaged using the Zeiss Observer Z.1 microscope using ZEN 3.7 software (Carl Zeiss Microimaging GmbH).

### Statistical analyses

Statistical analyses were performed in GraphPad Prism 9.1.0 (GraphPad Software, Inc., San Diego, CA). Results are reported as mean ± standard deviation. Statistical assessment was carried out by one- or two-way analysis of variance (ANOVA) with Šídák’s or Tukey’s post hoc test for multiple comparisons; ns = non-significant, *p < .05, **p < .01, ***p < .001.

## References

[1] FDA. FDA grants accelerated approval for Alzheimer’s disease treatment. 2023 [accessed 2023 Jan 6 https://www.fda.gov/news-events/press-announcements/fda-grants-accelerated-approval-alzheimers-disease-treatment.

[2] Englund H, Sehlin D, Johansson A-S, Nilsson LNG, Gellerfors P, Paulie S, Lannfelt L, Pettersson FE. Sensitive ELISA detection of amyloid-β protofibrils in biological samples. J Neurochem. 2007;103(1):334–11. doi:10.1111/j.1471-4159.2007.04759.x. 17623042

[3] van Dyck CH, Swanson CJ, Aisen P, Bateman RJ, Chen C, Gee M, Kanekiyo M, Li D, Reyderman L, Cohen S. et al. Lecanemab in early Alzheimer’s disease. N Engl J Med. 2023;388(1):9–21. doi:10.1056/NEJMoa2212948. 36449413

[4] Cavazzoni P. (FDA C. for D. E. and R. FDA’s decision to approve new treatment for Alzheimer’s disease. *Fda’s decision to approve new treatment for Alzheimer’s disease* 2021. https://www.fda.gov/drugs/news-events-human-drugs/fdas-decision-approve-new-treatment-alzheimers-disease.

[5] Sevigny J, Chiao P, Bussière T, Weinreb PH, Williams L, Maier M, Dunstan R, Salloway S, Chen T, Ling Y. et al. The antibody aducanumab reduces Aβ plaques in Alzheimer’s disease. Nature. 2016;537(7618):50–56. doi:10.1038/nature19323. 27582220

[6] Gustavsson T, Metzendorf NG, Wik E, Roshanbin S, Julku U, Chourlia A, Nilsson P, Andersson KG, Laudon H, Hultqvist G. et al. Long-term effects of immunotherapy with a brain penetrating Aβ antibody in a mouse model of Alzheimer’s disease. Alzheimers Res Ther. 2023;15(1):90. doi:10.1186/s13195-023-01236-3. 37131196 PMC10152635

[7] Klunk WE, Engler H, Nordberg A, Wang Y, Blomqvist G, Holt DP, Bergström M, Savitcheva I, Huang G-F, Estrada S. et al. Imaging brain amyloid in Alzheimer’s disease with Pittsburgh Compound-B. Ann Neurol. 2004;55(3):306–19. doi:10.1002/ana.20009. 14991808

[8] Schilling LP, Zimmer ER, Shin M, Leuzy A, Pascoal TA, Benedet AL, Borelli WV, Palmini A, Gauthier S, Rosa-Neto P. Imaging Alzheimer’s disease pathophysiology with PET. Dementia e Neuropsychologia. 2016;10(2):79–90. Preprint at. doi:10.1590/S1980-5764-2016DN1002003. PMC5642398 29213438

[9] Glabe CG, Kayed R. Common structure and toxic function of amyloid oligomers implies a common mechanism of pathogenesis. Neurology. 2006;66(1_suppl_1):S74–78. doi:10.1212/01.wnl.0000192103.24796.42. 16432151

[10] Massoud TF, Gambhir SS. Molecular imaging in living subjects: seeing fundamental biological processes in a new light. Genes Dev. 2003;17(5):545–80. doi:10.1101/gad.1047403. 12629038

[11] Sehlin D, Syvänen S. Engineered antibodies: new possibilities for brain PET? Eur J Nucl Med Mol Imaging. 2019;11(13):2848–58. doi:10.1007/s00259-019-04426-0. PMC6879437 31342134

[12] Lord A, Gumucio A, Englund H, Sehlin D, Sundquist VS, Söderberg L, Möller C, Gellerfors P, Lannfelt L, Pettersson FE. et al. An amyloid-β protofibril-selective antibody prevents amyloid formation in a mouse model of Alzheimer’s disease. Neurobiol Dis. 2009;36(3):425–34. doi:10.1016/j.nbd.2009.08.007. 19703562

[13] Hultqvist G, Syvänen S, Fang XT, Lannfelt L, Sehlin D. Bivalent brain shuttle increases antibody uptake by monovalent binding to the transferrin receptor. Theranostics. 2017;7(2):308–18. doi:10.7150/thno.17155. 28042336 PMC5197066

[14] Sehlin D, Fang XT, Cato L, Antoni G, Lannfelt L, Syvänen S. Antibody-based PET imaging of amyloid beta in mouse models of Alzheimer’s disease. Nat Commun. 2016;7(1):1–11. doi:10.1038/ncomms10759. PMC4762893 26892305

[15] Yu YJ, Atwal JK, Zhang Y, Tong RK, Wildsmith KR, Tan C, Bien-Ly N, Hersom M, Maloney JA, Meilandt WJ. et al. Therapeutic bispecific antibodies cross the blood-brain barrier in nonhuman primates. Sci Transl Med. 2014;6(261). doi:10.1126/scitranslmed.3009835. 25378646

[16] Rofo F. Meier, SR, Metzendorf, NG, Morrison, JI, Petrovic A, Syvänen S, Sehlin D, Hultqvist G. A brain-targeting bispecific-multivalent antibody clears soluble amyloid-beta aggregates in Alzheimer’s Disease Mice. Neurotherapeutics. 2022;19:1588–602. doi:10.1007/s13311-022-01283-y. 35939261 PMC9606191

[17] Rofo F, Ugur Yilmaz C, Metzendorf N, Gustavsson T, Beretta C, Erlandsson A, Sehlin D, Syvänen S, Nilsson P, Hultqvist G. et al. Enhanced neprilysin-mediated degradation of hippocampal Aβ42 with a somatostatin peptide that enters the brain. Theranostics. 2020;11(2):789–804. doi:10.7150/thno.50263. PMC7738863 33391505

[18] Kariolis MS, Wells RC, Getz JA, Kwan W, Mahon CS, Tong R, Kim DJ, Srivastava A, Bedard C, Henne KR. et al. Brain delivery of therapeutic proteins using an Fc fragment blood-brain barrier transport vehicle in mice and monkeys. Sci Transl Med. 2020;12(545):eaay1359. doi:10.1126/scitranslmed.aay1359. 32461332

[19] Boado RJ, Zhang Y, Wang Y, Pardridge WM. Engineering and expression of a chimeric transferrin receptor monoclonal antibody for blood–brain barrier delivery in the mouse. Biotechnol Bioeng. 2009;102(4):1251–58. doi:10.1002/bit.22135. 18942151 PMC2729652

[20] Sehlin D, Fang XT, Meier SR, Jansson M, Syvänen S. Pharmacokinetics, biodistribution and brain retention of a bispecific antibody-based PET radioligand for imaging of amyloid-β. Sci Rep. 2017;7(1):1–9. doi:10.1038/s41598-017-17358-2. 29222502 PMC5722892

[21] Niewoehner J, Bohrmann B, Collin L, Urich E, Sade H, Maier P, Rueger P, Stracke J, Lau W, Tissot A. et al. Increased brain penetration and potency of a therapeutic antibody using a monovalent molecular shuttle. Neuron. 2014;81(1):49–60. doi:10.1016/j.neuron.2013.10.061. 24411731

[22] Morrison JI, Metzendorf NG, Rofo F, Petrovic A, Hultqvist G. A single-chain fragment constant design enables easy production of a monovalent blood–brain barrier transporter and provides an improved brain uptake at elevated doses. J Neurochem. 2023;165(3):413–25. doi:10.1111/JNC.15768. 36681883

[23] Yu YJ, Zhang Y, Kenrick M, Hoyte K, Luk W, Lu Y, Atwal J, Elliott JM, Prabhu S, Watts RJ. et al. Boosting brain uptake of a therapeutic antibody by reducing its affinity for a transcytosis target. Sci Transl Med. 2011;3(84). doi:10.1126/scitranslmed.3002230. 21613623

[24] Montoyo HP, Vaccaro C, Hafner M, Ober RJ, Mueller W, Ward ES. Conditional deletion of the MHC class l-related receptor FcRn reveals the sites of IgG homeostasis in mice. Proc Natl Acad Sci U S A. 2009;106(8):2788–93. doi:10.1073/pnas.0810796106. 19188594 PMC2650344

[25] Challa DK, Wang X, Montoyo HP, Velmurugan R, Ober RJ, Ward ES. Neonatal Fc receptor expression in macrophages is indispensable for IgG homeostasis. Mabs-austin. 2019;11(5):848–60. doi:10.1080/19420862.2019.1602459. PMC6601554 30964743

[26] Cooper PR, Ciambrone GJ, Kliwinski CM, Maze E, Johnson L, Li Q, Feng Y, Hornby PJ. Efflux of monoclonal antibodies from rat brain by neonatal Fc receptor, FcRn. Brain Res. 2013;1534:13–21. doi:10.1016/j.brainres.2013.08.035. 23978455

[27] Schlachetzki F, Zhu C, Pardridge WM. Expression of the neonatal Fc receptor (FcRn) at the blood–brain barrier. J Neurochem. 2002;81(1):203–06. doi:10.1046/j.1471-4159.2002.00840.x. 12067234

[28] Garg A, Balthasar JP. Investigation of the influence of FcRn on the distribution of IgG to the brain. Aaps J. 2009;11(3):553–57. doi:10.1208/s12248-009-9129-9. 19636712 PMC2758122

[29] Finke JM, Banks WA, Steinitz M. Modulators of IgG penetration through the blood-brain barrier: Implications for Alzheimer’s disease immunotherapy. Hum Antibodies. 2017;25(3–4):131–46. doi:10.3233/HAB-160306. 28035915

[30] Zhang Y, Pardridge WM. Mediated efflux of IgG molecules from brain to blood across the blood–brain barrier. J Neuroimmunol. 2001;114(1–2):168–72. doi:10.1016/S0165-5728(01)00242-9. 11240028

[31] Nazarova L, Rafidi H, Mandikian D, Ferl GZ, Koerber JT, Davies CW, Ulufatu S, Ho J, Lau J, Yu S-F. et al. Effect of modulating FcRn binding on direct and pretargeted tumor uptake of full-length antibodies. Mol Cancer Ther. 2020;19(4):1052–58. doi:10.1158/1535-7163.MCT-19-1015. 32024685

[32] Swiercz R, Chiguru S, Tahmasbi A, Ramezani SM, Hao G, Challa DK, Lewis MA, Kulkarni PV, Sun X, Ober RJ. et al. Use of Fc-engineered antibodies as clearing agents to increase contrast during PET. J Nucl Med. 2014;55(7):1204–07. doi:10.2967/jnumed.113.136481. 24868106 PMC4519079

[33] Vanoli F, Mantegazza R. Antibody therapies in autoimmune neuromuscular junction disorders: Approach to myasthenic crisis and chronic management. Neurotherapeutics. 2022;19(3):897–910. doi:10.1007/s13311-022-01181-3. 35165857 PMC9294078

[34] Vilhelmsson Timmermand O, Örbom A, Altai M, Zedan W, Holmqvist B, Safi M, Tran TA, Strand S-E, Strand J. A conjugation strategy to modulate antigen binding and FcRn interaction leads to improved tumor targeting and radioimmunotherapy efficacy with an antibody targeting prostate-specific antigen. Cancers Basel. 2021;13(14):3469. doi:10.3390/cancers13143469. 34298682 PMC8307315

[35] Rudnik-Jansen I, Howard KA. FcRn expression in cancer: Mechanistic basis and therapeutic opportunities. J Control Release. 2021;337:248–57. doi:10.1016/j.jconrel.2021.07.007. 34245786

[36] Saunders KO. Conceptual approaches to modulating antibody effector functions and circulation half-life. Front Immunol. 2019;10:1296. doi:10.3389/fimmu.2019.01296. 31231397 PMC6568213

[37] Greenwood C, Hunter WM, Glover JS, Marsh, CA. The preparation of 131I-Labelled human growth hormone of high specific radioactivity. J Chem Soc. 1963;227(1):114–123. doi:10.1042/bj0890114. PMC1202279 14097352

[38] Miles LA, Crespi GAN, Doughty L, Parker MW. Bapineuzumab captures the N-terminus of the Alzheimer’s disease amyloid-beta peptide in a helical conformation. Sci Rep. 2013;3(1). doi:10.1038/srep01302. PMC3575012 23416764

[39] Kissel K, Hamm S, Schulz M, Vecchi A, Garlanda C, Engelhardt B. Immunohistochemical localization of the murine transferrin receptor (TfR) on blood–tissue barriers using a novel anti-TfR monoclonal antibody. Histochem Cell Biol. 1998;110(1):63–72. doi:10.1007/s004180050266. 9681691

[40] Robin B, Games-Thiel, KD, Niijar, TS, Zago W, Mundigl O, Niewoehner J, Tiefenthaler G, inventors; Prothena Biosciences Ltd, Hoffmann La Roche Inc, assignee. Blood-brain barrier shuttles containing antibodies recognizing alpha-synuclein. 2017.

[41] Lo M, Kim HS, Tong RK, Bainbridge TW, Vernes J-M, Zhang Y, Lin YL, Chung S, Dennis MS, Zuchero YJY. et al. Effector-attenuating substitutions that maintain antibody stability and reduce toxicity in mice. J Biol Chem. 2017;292(9):3900–08. doi:10.1074/jbc.M116.767749. 28077575 PMC5339770

[42] Bonvicini G, Bagawath Singh S, Nygren P, Xiong M, Syvänen S, Sehlin D, Falk R, Andersson KG. Comparing in vitro affinity measurements of antibodies to TfR1: Surface plasmon resonance versus on-cell affinity. Anal Biochem. 2023;686:115406. doi:10.1016/j.ab.2023.115406. 38006952

[43] Syvänen S, Hultqvist G, Gustavsson T, Gumucio A, Laudon H, Söderberg L, Ingelsson M, Lannfelt L, Sehlin D. Efficient clearance of Aβ protofibrils in AβPP-transgenic mice treated with a brain-penetrating bifunctional antibody. Alzheimers Res Ther. 2018;10(1). doi:10.1186/s13195-018-0377-8. PMC5968497 29793530

[44] Gustavsson T, Syvänen S, O’callaghan P, Sehlin D. SPECT imaging of distribution and retention of a brain-penetrating bispecific amyloid-β antibody in a mouse model of Alzheimer’s disease. Transl Neurodegener. 2020;9(1):1–11. doi:10.1186/s40035-020-00214-1. 32951598 PMC7504681

[45] Rees AV. JCR pharmaceuticals announces approval of IZCARGOⓇ (Pabinafusp Alfa) for Treatment of MPS II (Hunter Syndrome) in Japan. 2021. https://www.businesswire.com/news/home/20210323005577/en/JCR-Pharmaceuticals-Announces-Approval-of-IZCARGOⓇ-Pabinafusp-Alfa-for-Treatment-of-MPS-II-Hunter-Syndrome-in-Japan.

[46] Tien J, Leonoudakis D, Petrova R, Trinh V, Taura T, Sengupta D, Jo L, Sho A, Yun Y, Doan E. et al. Modifying antibody-FcRn interactions to increase the transport of antibodies through the blood-brain barrier. Mabs-austin. 2023;15(1):1–12. doi:10.1080/19420862.2023.2229098. PMC10312019 37381177

[47] Pizzo ME, Wolak DJ, Kumar NN, Brunette E, Brunnquell CL, Hannocks M-J, Abbott NJ, Meyerand ME, Sorokin L, Stanimirovic DB. et al. Intrathecal antibody distribution in the rat brain: surface diffusion, perivascular transport and osmotic enhancement of delivery. Journal Of Physiol J. 2018;596(3):445–75. doi:10.1113/JP275105. PMC5792566 29023798

[48] Ober RJ, Radu CG, Ghetie V, Ward ES. Differences in promiscuity for antibody–FcRn interactions across species: implications for therapeutic antibodies. Int Immunol. 2001;13(12):1551–59. doi:10.1093/intimm/13.12.1551. 11717196

[49] Saito T, Matsuba Y, Mihira N, Takano J, Nilsson P, Itohara S, Iwata N, Saido TC. Single app knock-in mouse models of Alzheimer’s disease. Nat Neurosci. 2014;17(5):661–63. doi:10.1038/nn.3697. 24728269

[50] Meier SR, Syvänen S, Hultqvist G, Fang XT, Roshanbin S, Lannfelt L, Neumann U, Sehlin D. Antibody-based in vivo PET imaging detects amyloid-β reduction in Alzheimer transgenic mice after BACE-1 inhibition. J Nucl Med. 2018;59(12):1885–91. doi:10.2967/jnumed.118.213140. 29853653 PMC6278900

[51] Meier SR, Sehlin D, Roshanbin S, Falk VL, Saito T, Saido TC, Neumann U, Rokka J, Eriksson J, Syvänen S. et al. 11 C-PiB and 124 I-Antibody PET provide differing estimates of brain amyloid-β after therapeutic intervention. J Nucl Med. 2022;63(2):302–09. doi:10.2967/jnumed.121.262083. 34088777 PMC8805773

[52] Faresjö R, Bonvicini G, Fang XT, Aguilar X, Sehlin D, Syvänen S. Brain pharmacokinetics of two BBB penetrating bispecific antibodies of different size. Fluids Barriers CNS. 2021;18(1):26. doi:10.1186/s12987-021-00257-0. 34078410 PMC8170802

[53] Lopes van den Broek S, Shalgunov V, García Vázquez R, Beschorner N, Bidesi NSR, Nedergaard M, Knudsen GM, Sehlin D, Syvänen S, Herth MM. et al. Pretargeted imaging beyond the blood–brain barrier–Utopia or Feasible? Pharmaceuticals (Basel). 2022;15(10):1191. doi:10.3390/ph15101191. 36297303 PMC9612205

[54] Schlein E, Syvänen S, Rokka J, Gustavsson T, Rossin R, Robillard M, Eriksson J, Sehlin D. Functionalization of radiolabeled antibodies to enhance peripheral clearance for high contrast brain imaging. Mol Pharm. 2022;19(11):4111–22. doi:10.1021/acs.molpharmaceut.2c00536. 36201682 PMC9644377

[55] Wuensche TE, Stergiou N, Mes I, Verlaan M, Schreurs M, Kooijman EJM, Janssen B, Windhorst AD, Jensen A, Asuni AA. et al. Advancing 89Zr-immuno-PET in neuroscience with a bispecific anti-amyloid-beta monoclonal antibody – the choice of chelator is essential. Theranostics. 2022;12(16):7067–79. doi:10.7150/thno.73509. 36276653 PMC9576608

